# Anti-Inflammatory Effect of Dialyzable Leukocyte Extract in Autoimmune Prostatitis: Evaluation in Animal Model

**DOI:** 10.1155/2017/1832853

**Published:** 2017-03-12

**Authors:** Carlos Pérez-Alvarado, Consuelo Gómez, Miguel Reyes, Mario García, Elizabeth Pérez, Carlos Pérez de la Mora, Virginia Sanchez, D. Guillermo Pérez Ishiwara

**Affiliations:** ^1^ENMH, Instituto Politécnico Nacional, Ciudad de México, Mexico; ^2^CBG, Instituto Politécnico Nacional, Cd. Reynosa, TAMPS, Mexico; ^3^Hospital General Milpa Alta, Ciudad de México, Mexico; ^4^Hospital de Traumatología, IMSS, Ciudad de México, Mexico; ^5^Farmainmune, Ciudad de México, Mexico

## Abstract

*Objective.* To evaluate the anti-inflammatory properties of Dialyzable Leukocyte Extract (DLE) in a murine model of chronic prostatitis/chronic pelvic pain syndrome (CP/CPPS).* Methods.* Histopathological characterization, prostatein Enzyme-Linked Immunosorbent Assay, and immunohistochemical analysis for CD45, TNF-*α*, IFN-*γ*, IL-6, IL-17, and IL-4 molecules were done in prostatic Wistar rats treated with DLE, placebo, or Dexamethasone.* Results. *Histopathological analysis of animals induced to prostatitis showed inflammatory infiltrate, mainly constituted by leucocytes and mast cells as well as Benign Prostatic Hyperplasia. Serum prostatein concentrations were 14 times higher than those displayed by healthy animals. After DLE and Dexamethasone treatments, the inflammatory infiltrate decreased; the tissue morphology was similar to that of a normal prostate, and the prostatein decreased to the basal levels of healthy animals. DLE treatment produced a decreased expression of the cell surface marker CD45 and the proinflammatory cytokines TNF-*α*, IFN-*γ*, IL-6, and IL-17. On the other hand, the expression of anti-inflammatory cytokine IL-4 increased in both the Dexamethasone and DLE groups.* Conclusion.* DLE is able to modulate the inflammatory response in Experimental Autoimmune Prostatitis (EAP).

## 1. Introduction

Prostatitis affects 10–20% of men from all ages and ethnic groups. The chronic prostatitis/chronic pelvic pain syndrome (CP/CPPS) comprises more than 90% of all cases [1, 2]. The etiological mechanism remains unclear, with complex clinical symptoms and recurrent episodes. Several authors suggest that CP/CPPS could be related to an autoimmune response [1]. Ludwig et al. have reported that T and B cells, granulocytes, and macrophages were found in semen and other expressed prostate secretions from CP/CPPS patients without infection [3]. On the other hand, Maccioni et al. found self-antigen specific IgG in sera from rats with CP/CPPS [4]. Furthermore, in a murine model of prostatitis, prostate-specific autoantibodies as well as NO, TNF-*α*, and IFN-*γ* in seminal plasma were elevated [5]. All these data suggest that CP/CPPS may have, in part, autoimmune etiopathogenic features that involve an immune response against prostate-specific antigens, thereby tending to prolong a chronic inflammation status [5, 6]. The constant inflammation in chronic prostatitis could be related to Benign Prostatic Hyperplasia (BPH) development or even more to prostatic cancer (PC) [1].

Prostatitis therapies have been based on the use of antibiotics and anti-inflammatory drugs, although alpha-blockers, 5-alpha reductase inhibitors, and some immunotherapeutic agents have also been used [1, 2]. Due to the undesirable side effects caused by conventional prostatitis treatments, many researchers have sought other agents that could regulate the immune response. For example, Elocalcitol, a vitamin D receptor agonist, decreases the inflammatory process by diminishing the expression of IFN-*γ* and IL-17 and by inhibiting CD4+ and CD8+ prostate infiltrate in a murine model [7]. Dialyzable Leukocyte Extract (DLE) has been used for the treatment of many inflammatory diseases, like osteoarthritis [8], autoimmune [9], bacterial [10], and viral [11] diseases among others. Pizza et al. in 1996 showed that DLE promoted cancer remissions and increased the survival of prostatic cancer patients [12]. Here, we report using a modified Experimental Autoimmune Prostatitis model [13], the effects of DLE treatment in autoimmune prostatitis through histopathological analysis, the detection of prostatein by Enzyme-Linked Immunosorbent Assay (ELISA), and immunohistochemical staining for anti- and proinflammatory cytokines.

## 2. Material and Methods

### 2.1. Animals

Three-month-old male Wistar rats were maintained under specific pathogen-free conditions at room temperature and a 12 h/12 h light/dark cycle. Experimental procedures with the animals were approved by the Bioethics and Biosecurity Committee from Escuela Nacional de Medicina y Homeopatía, IPN.

### 2.2. Prostatic Antigens Preparation

Male sex rat accessory glands (RAG) were excised from ten rats and homogenized in a Pyrex homogenizer by using 0.01 M PBS at pH 7.2, in the presence of protease inhibitors (Roche 0469311600 Complete; Mannheim, Germany). The homogenate was centrifuged at 10000*g* for 30 min and the supernatant was then used as RAG homogenate. Finally, the RAG homogenate was chemically modified (MRAG) by coupling the RAG saline extract to a diazonium derivate of sulphanilic (0.0865 g) and arsanilic (0.1085 g) acids.

### 2.3. Immunization and Treatments

Rats (*n* = 25) were MRAG intradermally injected at day 0; then, the animals received intraperitoneal injections at day 15, followed by another intradermal injection at day 30. The immunizations were done with 5 mg of MRAG emulsified with 0.5 ml of Freund's complete adjuvant (FCA). After the second immunization (15 days), groups of 5 animals were formed and treated by oral administration with DLE, dialyzable leucocyte extract from* Crocodylus moreletii* [8], (0.133  mg/Kg/day) (DLE group), Dexamethasone (0.15 mg/kg/day) (Dexamethasone group), or Glycine (50 mg/kg/day) (Placebo group), daily during 34 days. A control group with the pathology without any treatment (WOT group) and a healthy group of rats that did not receive any kind of manipulation (normal group) were included. All the rats were sacrificed at day 34 to obtain prostatic tissue and serum.

### 2.4. Histopathological Analyses

The prostates of animals from all groups were dissected and sections were fixed in 10% formalin, dehydrated in alcohol, cleared in xylol, and embedded in paraffin. Five *µ*m sections were cut and stained with hematoxylin-eosin or toluidine blue at pH 3.5. Slides were examined and scored by 2 experienced pathologists, independently. Inflammatory cells were counted in 10 aleatory fields under high-power fields (HPF, 400x magnification), and the values of 5 slides per group were averaged.

### 2.5. ELISA

Serum prostatein concentrations were determined by using ELISA kit assay (SEE174Hu; USCN, Wuhan, China) according to the manufacturer's instructions. Briefly, multiwall plates were coated with 100 *µ*l per well of LIPB standard solution or serum samples obtained from animals, incubated at 37°C for 2 h and washed with washing solution. Solution A was added, incubated at 37°C for 1 h, and washed again. Then, solution B was added, incubated at 37°C for 30 min, and washed. Ninety *µ*l of substrate solution was added and incubated at 37°C for 30 min. Finally, 50 *µ*l of stop solution was added. Absorbances were measured at 450 nm. The samples were analyzed in triplicate and the mean concentrations were calculated. The sensitivity limit of the method was 0.114 ng/mL.

### 2.6. Immunohistochemistry Assays

Prostatic sections were fixed overnight in a 10% buffered paraformaldehyde solution. The tissues were ethanol-dehydrated and embedded in paraffin wax. Sections of 5 *µ*m were cut with microtome, deparaffinized at 70°C for 20 min, incubated 5 minutes each in xylene, ethanol-xylene, and 100% ethanol. Finally, the samples were washed with distilled water to be processed for immunohistochemical analysis. Immunolabeling was done by using Mouse/Rabbit PolyDetector Plus HRP/DAB (BSB 0259; BioSB) Kit according to the manufacturer's instructions. The antibodies used were TNF-*α* (50 *µ*g/mL) (Abcam 1793; Massachusetts, USA), IFN-*γ* (50 *µ*g/mL) (Abcam 7740; Massachusetts, USA), CD45 (1 *µ*g/mL) (Abcam 10558, Massachusetts, USA), IL-17 (1 *µ*g/mL) (Abcam 79056, Massachusetts, USA), IL-6 (74 *µ*g/mL) (Abcam 6672; Massachusetts, USA), IL-4 (0.5 *µ*g/Ml) (Abcam 11524; Massachusetts, USA), and PCNA (100 *µ*g/mL) (Santa Cruz-53407, Texas, USA). After the immunohistochemistry procedure, microphotographs were taken and the relative expression percentages were semiquantified in the Image-Pro Premier software 9.1.

### 2.7. Statistical Analysis

Data were expressed as the mean ± standard deviation (SD) of the indicated numbers of samples. One-way ANOVA and Tukey's multiple comparison test were used to assess changes in the levels of prostatein and in the expression percentage of the different cytokines. Two-tailed tests were used for all processes, and *p* values ≤ 0.05 were considered statistically significant. The analysis was done with GraphPad Prism 6.

## 3. Results

### 3.1. DLE Decreases BPH and Inflammatory Cells

Hematoxylin-eosin staining and histological analysis of the prostate showed few changes in prostatic urethra, showing only a little urothelial metaplasia of prostatic acinar epithelium and a faint fibrosis in the prostatic area adjacent to the seminal vesicles (data not shown). However, in the subcapsular peripheral zone we observed the histological and morphological changes, such as inflammatory infiltration and hyperplasia, so we decided to focus our analysis in this area.

**Figure 1:**
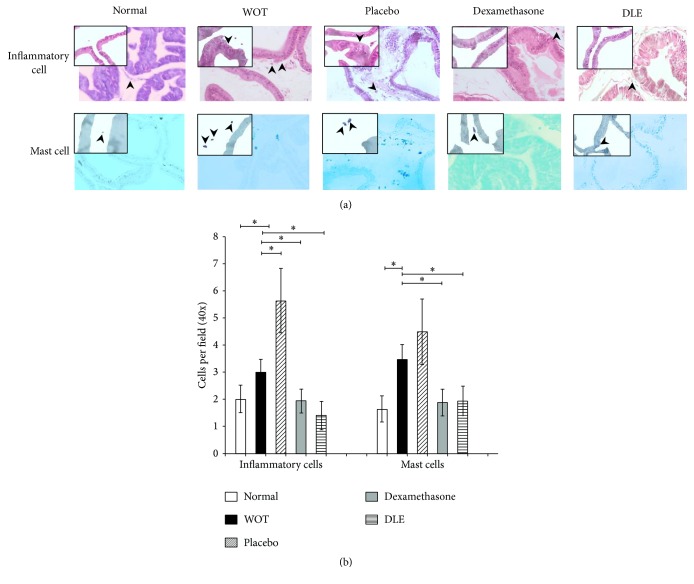
DLE decreases the inflammatory and mast cell infiltration. (a) Animals from the placebo and WOT groups showed a notorious infiltrate of inflammatory and mast cells. Also, tissue regions with prostatic intraepithelial atrophy and BPH were observed. Microphotographs 40x. Inset close-up 100x. (b) Quantification of the inflammatory and mast cells. “*∗*” indicates groups that showed statistical significance (*P* < 0.05), and the lines indicate groups that were compared.

In nonpathological group hematoxylin-eosin staining showed that this group displayed normal prostatic histology characterized by alveolar glands of regular size and form, a clear basal membrane lined by a tall columnar epithelium (Figure 1(a)). Few inflammatory and mast cells were observed (approximately 2 cells per HPF) (Figure 1(b)). In the pathological group without treatment (WOT), or in the placebo group, we observed hyperplasia of glandular structures with a thickened basal membrane, coated with a cellular hypertrophic epithelium (Figure 1(a)). Besides, an infiltrate of inflammatory cells, from 3.03 to 5.64 cells per HPF, respectively, was found (Figure 1(b)). The presence of inflammatory cells in the WOT and placebo groups was significantly higher in comparison with normal group. On the other hand, prostatic tissue from the Dexamethasone treated group showed slightly hypertrophic epithelium and glands with a thick basal membrane and edema in the extracellular matrix, with an infiltrate of 1.94 cells per HPF on average. In DLE treated group the architecture of the prostatic tissue showed glands with a regular size and shape with a thin basal cell membrane similar to the tissue of normal animals and with 1.38 inflammatory cells per HPF on the average. Cellular infiltrate in the Dexamethasone and DLE treated groups was statistically lower than in the WOT and placebo treated ones.

Toluidine blue staining showed that in the nonpathological group the amount of mast cells was 1.64 cells per HPF, while in the WOT and placebo treated groups there were 4.49 and 3.47 cells per HPF, respectively. Instead, the DLE and Dexamethasone treated groups showed a decreased amount of mast cells compared to the WOT group, displaying an average of 1.88 and 1.93 cells per HPF, respectively (Figure 1(b)). Statistical analysis showed that the decrease in mast cell numbers was significant in the DLE and Dexamethasone groups in contrast with the WOT and placebo groups (Figure 1(b)).

### 3.2. The DLE Decrease the Prostatein Serum Levels

**Figure 2:**
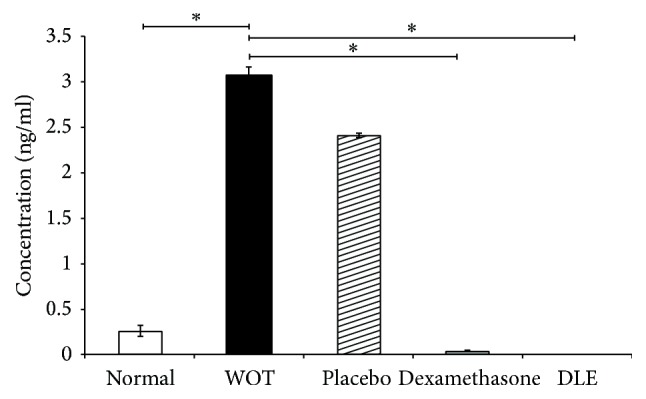
DLE decreased the prostatein serum concentration of prostatein in comparison with other groups. The serum level of prostatein in the Dexamethasone group also diminished. The placebo and WOT group presented a significantly higher prostatein concentration. “*∗*” indicates groups that showed statistical significance (*P* < 0.05), and the lines indicate groups that were compared.

Prostatein serum concentrations showed that the normal concentration in nonpathological animals was 0.22 ng/ml (±0.06). Whereas WOT animals or animals with the pathology treated with placebo displayed 3.1 ng/ml and 2.4 ng/ml, respectively, representing 14.09- and 10.9-fold excess, over healthy animals. In contrast, the groups treated with Dexamethasone and interestingly those treated with DLE displayed an important reduction, 0.04 ng/ml or nondetectable concentrations, respectively (Figure 2).

### 3.3. DLE Decreases Cell Proliferation

**Figure 3:**
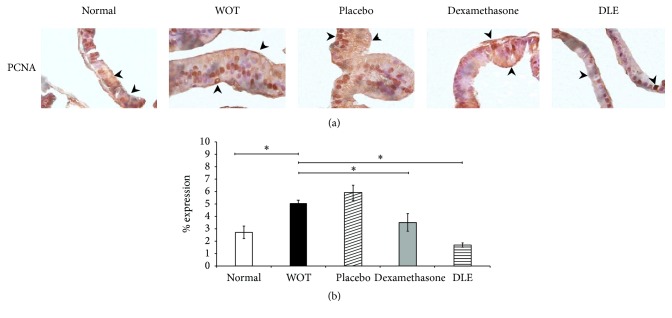
DLE decreased the expression of PCNA. (a) Microphotographs are in 100x. Normal tissue showed an expression of PCNA similar to the Dexamethasone group. The DLE group presented a decreased expression of PCNA in comparison with the other treatments. The placebo and WOT groups presented a significantly higher expression of PCNA in contrast to other groups. (b) Semiquantification of PCNA expression. “*∗*” indicates groups that showed statistical significance (*P* < 0.05), and the lines indicate groups that were compared.

Figure 3 shows the immunohistochemical analysis of the PCNA cell proliferation marker. In normal tissue we observed a clear basal membrane constituted only by a lined tall columnar epithelium; in contrast, in the WOT and placebo groups we observed a pseudostratified epithelium and hyperplasia (Figure 3(a)). On the other hand in the Dexamethasone and DLE groups we noted a monolayer of columnar epithelium similar to that in the normal group (Figure 3(a)). Semiquantification of the PCNA marker indicated that in the normal group we observed a 2.72% expression rate; in contrast, animals from the WOT and placebo groups displayed 5.03% and 5.9%, respectively (Figure 3(b)). On the other hand, the Dexamethasone group displayed a PCNA expression rate of 3.5%, while animals from the DLE group showed a diminished PCNA expression (1.7%) which is statistically different in contrast to the WOT group (Figure 3(b)).

### 3.4. DLE Decreased the Expression of CD45, TNF-*α*, IFN-*γ*, IL-6, and IL-17

**Figure 4:**
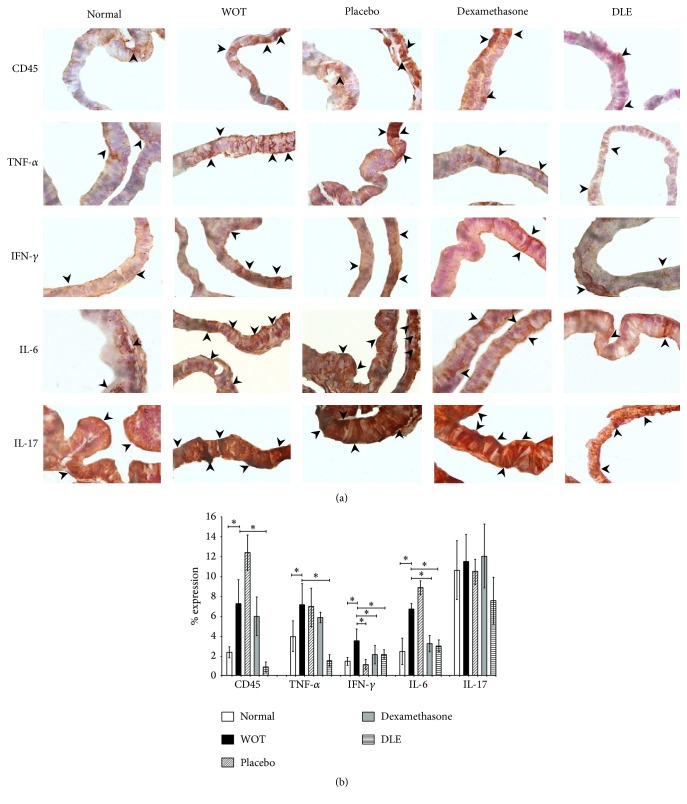
DLE modified the expression of some proinflammatory molecules. The expression percentage was semiquantified with software Image-Pro Premier. (a) Microphotographs are in 100x. CD45 in normal tissue showed a basal expression. CD45 expression in the Dexamethasone treated group was similar to the WOT group. The DLE treated group showed a significantly lesser expression in comparison to the other groups. TNF-*α* in the normal group also showed a basal expression. The Dexamethasone treated group showed a TNF-*α* expression similar to the WOT. The DLE treated group showed a significantly lower expression than that in other groups. IFN-*γ* in the normal group displayed a basal expression. The placebo treated group showed a decreased IFN-*γ* expression in comparison with the other groups. The Dexamethasone and DLE treated groups showed an IFN-*γ* expression similar to normal group. IL-6 in the normal group showed a basal expression. In the Dexamethasone and DLE treated groups, the IL-6 expression was similar to the normal group. For IL-17 we did not observe any significant differences. (b) Graph of the semiquantification of the percentage of expression in all groups. “*∗*” indicates groups that showed statistical significance (*P* < 0.05), and the lines indicate groups that were compared.

CD45 is located in leucocytes and some investigations also reported that prostatic cells could express it [14]. In human BPH, Adegun et al., 2013, found CD45 in the epithelium of the gland, staining it very strongly [14]. Here we found that in a normal epithelium we observed a low signal of CD45 in the apical region of the gland (Figure 4(a)). The WOT and placebo groups showed an intense immunohistochemical signal in the basal and luminal region (Figure 4(a)). The signal of this marker in the Dexamethasone and DLE treated groups was diffuse in the apical glandular region and like the one we observed in the normal group (Figure 4(a)). Making a distribution analysis along ten aleatory zones in prostatic tissue, we found a basal staining rate (2.34%) in nonpathological animals, while animals with prostatitis WOT or from placebo groups displayed 7.25% and 12.36% (*P* < 0.05), respectively (Figure 4(b)). The animals from the Dexamethasone treated group did not show a significant difference in the CD45 rate of expression (5.94%) when we compare them with those from the WOT group. In contrast, the animals treated with DLE displayed a statistically significant reduction in the CD45 expression (0.86%) (Figure 4(b)).

TNF-*α* is expressed in the cell membrane of T cells, fibroblasts, macrophages, and mast cells and could spread to the glandular epithelium [15]. Sugihara et al. showed that TNF-*α* was detected by immunohistochemical staining in the cytoplasm of the epithelium of noncancerous prostatic human glands and also in epithelium of prostatic cancer glands [16]. We observed here in normal group a weak signal in the apical glandular region of the glandular epithelium (Figure 4(a)). The WOT group showed an intense but diffuse signal and in the placebo treated group the signal increased in the luminal and basal regions (Figure 4(a)). In the Dexamethasone and DLE treated groups the signal decreased in comparison with the signal presented in the WOT and placebo treated groups (Figure 4(a)). Semiquantification showed a 3.94% expression in the normal group, which was significantly lower than the expression showed in the WOT (7.11%) or in the placebo (6.91%) animals (Figure 4(b)). The Dexamethasone treated group had a 5.83% expression rate, which was increased statistically to that of the WOT group. The animals treated with DLE displayed a 1.5% expression rate, which was significantly lower than in the other groups (Figure 4(b)).

IFN-*γ* is expressed in T cells, mast cells, and fibroblasts and is also expressed in basal and epithelial prostatic cells [17]. Royuela et al., 2000, indicated that IFN-*γ* was also expressed by immunohistochemistry in basal cells of the prostatic epithelium and in some stromal cells. Its expression was higher in both basal and columnar epithelial cells in specimens with BPH and cancer [17]. In our study we observed that the normal group showed the signal for this molecule in the rim of the glandular epithelium (Figure 4(a)). In the WOT and placebo groups we observed a more intense but diffuse immunohistochemical signal in the luminal and basal glandular regions (Figure 4(a)). In the Dexamethasone and DLE treated groups we noted a diffuse and weak signal of this marker in the luminal and basal regions (Figure 4(a)). In the semiquantification analysis, we observed in the normal group that the IFN-*γ* expression was of 1.4%, while in the WOT it was higher (3.5%), and in the placebo group the expression was 1.1% (Figure 4(b)). On the other hand, the groups treated with Dexamethasone and DLE showed similar expression (2.1%) with no statistical differences from the healthy group (Figure 4(b)).

IL-6 is expressed principally in T cells, fibroblast, macrophages, and mast cells; however, it could also be detected interacting with its receptor in prostatic cells [18]. Miličević et al. in 2015 showed that IL-6 is expressed in epithelial and basal prostatic cells from normal, prostatic, and BPH and in premalignant and malignant epithelial cells as well as in some stromal cells [19]. Here, the healthy group showed a spotted IL-6 signal distribution in basal and luminal regions (Figure 4(a)). In the WOT and placebo groups we observed an increased expression area, detecting a diffuse signal in basal and luminal regions (Figure 4(a)). On the other hand, the Dexamethasone and DLE treated groups showed a smaller area in basal and luminal regions (Figure 4(a)). Semiquantification showed an expression rate of 2.43% in the normal group. In contrast, the WOT and placebo groups displayed 6.72 and 8.82%, respectively (Figure 4(b)). However the application of Dexamethasone and DLE treatments diminished the IL-6 expression area to 3.23% and 2.97%, respectively (Figure 4(b)).

IL-17 is expressed in T cells and mast cells principally and could also interact with its receptor in prostatic cells [20]. Moseley and coworkers indicated that in current versions of the EST database some isoforms of IL-17 are expressed in the prostate [21]. Other authors also demonstrated by immunohistochemistry that IL-17 increases its expression in both stromal and epithelial tissue of the dorsolateral prostate in a murine prostatitis model [22]. In our case we observed in normal group an IL-17 signal in basal and luminal glandular regions (Figure 4(a)). The WOT and placebo groups were also showing an intense signal in said regions (Figure 4(a)). In the Dexamethasone group we again observed a signal of this marker in the basal and luminal region, whereas the DLE treated group showed the signal for IL-17 with a frosted pattern (Figure 4(a)). However, in the semiquantitative analysis of this molecule, we did not observe any significant differences of expression among the nonpathological (10.6%), WOT (11.49%), placebo (11.49%), Dexamethasone (12.03%), and DLE (7.52%) groups (*P* = 0.5) (Figure 4(b)).

### 3.5. DLE Increased the Expression of Anti-Inflammatory IL-4

**Figure 5:**
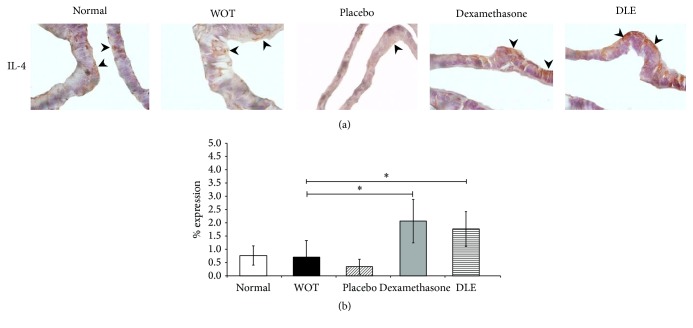
DLE increased the IL-4 expression. The expression percentage was semiquantified with software Image-Pro Premier. (a) Microphotographs are in 100x; the normal group showed a basal expression of IL-4, similar to that displayed by the placebo and WOT treated groups. The Dexamethasone and DLE groups displayed a significantly higher IL-4 expression than the other groups. (b) Semiquantification of the IL-4 expression in all groups. “*∗*” indicates groups that showed statistical significance (*P* < 0.05), and the lines indicate groups that were compared.

Finally, IL-4 is expressed in T cell and mast cells and it could also be detected associated with its receptor in prostatic cells [23]. Goldstein and coworkers, 2011, showed a different immunoreactivity of IL-4 in prostatic cells of BPH and prostatic cancer. Analysis of benign and malignant prostate tissues demonstrated that the source of IL-4 is the epithelial cells rather than infiltrating leukocyte [24]. In the normal group we observed a glandular epithelium with cellular monolayer and an IL-4 signal in the basal area, while in the WOT and placebo groups we observed hyperplasic and hypertrophic regions and a poor IL-4 expression in the luminal region (Figure 5(a)). In the Dexamethasone and DLE groups we observed glandular tissue morphology similar to that of the normal group and an increased IL-4 signal in luminal and basal glandular areas (Figure 5(a)). Semiquantitative analysis showed 0.76% of expression in the normal group which was statistically similar to that expressed in the WOT and placebo groups (0.76 and 0.36%, resp.) (Figure 5(b)). In contrast, in the groups treated with Dexamethasone (2.05%) and DLE (1.76%), we observed a significant increase in IL-4 expression (*P* < 0.05) (Figure 5(b)).

## 4. Discussion

Several authors have reported that DLE can modulate the immune response in several diseases, like bacterial diseases [10], autoimmune diseases [9], cancer [12, 25], and others [26–29]. The DLE effects on the immune system include cytokine modulation [27], monocyte activation, macrophage chemotaxis, and natural killer activity enhancement [11]. In vitro, DLE regulates the NF-*κ*B activity and reduces TNF-*α* and TGF-*B*1 secretion, downregulating the HIV transcription [11]. Recently, our group also showed that DLE diminishes the inflammatory and degenerative processes of cartilage by increasing the synthesis of proteoglycans, promoting collagen formation and the induction of tissue repair in a murine model of osteoarthritis [8]. DLE reduced the NF-*κ*B nuclear translocation, thus promoting the expression of the anti-inflammatory cytokines PDGF and FGF-2 [8]. These evidences strongly suggest that DLE regulates the inflammatory process in some diseases [8].

CP/CPPS is considered an autoimmune inflammatory pathology characterized by the presence of inflammatory cells such as granulocytes, macrophages, mast cells, and T and B lymphocytes in prostatic secretions or in prostatic tissue. The inflammation could be mediated by an adaptive immune response directed against a self-genital tract antigen, like prostatein [4, 7] and spermine [6]. Dexamethasone, a well-known NF-*κ*B inhibitor, decreased the leukocyte extravasation to the prostate and reduced the activity of proinflammatory cytokines, IL-1*β* and IL-17 [30]. Some antibiotics, like ciprofloxacin and lomefloxacin, have also been used for prostatitis treatment; therefore it is known that these drugs could have an anti-inflammatory effect, independently of their antimicrobial effects [31]. Other drugs conventionally used in chronic prostatitis, such as alpha-blockers like alfuzosin, have been employed, demonstrating little clinical improvement after several months of treatment [31]. Due to the undesirable secondary effects and the longer administration time of conventional drug treatments [32], several researchers have proposed other agents for the prostatic inflammation treatment, such as modulators of the immune system. For example,* Ginkgo biloba* leaf extract decreased the signs of inflammation associated with BPH in prostatic tissue [33]. Also, a clinical report on metastatic prostate cancer suggested a correlation between DLE treatment and patient survival or remission [12]. Here, we used a modification of the Galmarini prostatitis model [13, 34], in order to increase the amount of mast cells and to induce epithelial atrophy and BPH [34]. Our model was also characterized by increased levels of prostatein, increment of epithelium proliferation, and the expression of some proinflammatory cytokines, such as TNF-*α*, IFN-*γ*, and IL-6 in the epithelium. In general, the inflammatory cell infiltrate produced a proinflammatory microenvironment that probably induced stromal hyperproliferation and tissue remodeling with local hypoxia [35]. Other authors have pointed out that infiltrated mast cells secrete proinflammatory and nociceptive mediators including histamine, cytokines, and proteolytic enzymes, thereby promoting the perpetuation of inflammatory microenvironment and pelvic pain, playing an important role in prostatitis and in the pathogenesis of Benign Prostatic Hyperplasia [36, 37].

The increase of the inflammatory infiltrate we observed in this work has been related in other works to the expression of prostatein. Penna and coworkers using EAP NOD mice model observed that the immunization with synthetic peptides of prostatein was related to increased inflammatory infiltrate [38], and Maccioni and coworkers, using a similar model reported here, state that prostatein secreted to the seminal fluid could be able to elicit prostatitis [4]. This is in concordance with Rivero et al. in 2002, suggesting that prostatein administration in NOD mice prostatitis model produced the pathology, characterized by lymphocytic inflammatory lesions in prostatic tissue [39]. Therefore, prostatein could be considered an autoantigen that facilitates the perpetuation of inflammation in EAP [38, 39]. It is known that persistent inflammation is considered the pathogenic background of hyperproliferation in prostatic tissue, possibly as a consequence of autoimmune responses and the increased synthesis of proinflammatory molecules [1]. For example, studies realized in a murine model of chronic bacterial inflammation showed that prostatic inflammation is characterized by the infiltration of inflammatory cells and the presence of epithelial and stromal hyperplasia [40]. In a NOD mice model of EAP, it was observed that Dexamethasone could decrease the inflammatory infiltrate [7]. Similarly, we observed that DLE diminished the serum prostatein levels and decreased the inflammatory infiltrate, thereby recovering the normal morphology of prostatic tissue in the animals treated with this agent.

The inflammatory microenvironment accompanied by hyperproliferation observed in the WOT group is in concordance with the increased expression of CD45 cell surface marker and TNF-*α* and IFN-*γ* proinflammatory cytokines within the epithelium. CD45 is a molecule associated with activated leucocytes but can also be found in other cells, such as breast cells, fibrocytes, and prostatic epithelial cells [14]. Adegun and coworkers observed increased expression of CD45 around the epithelium of the prostatic gland in biopsies of BPH patients [14]. Similarly, we observed increased expression of CD45 in prostatic tissue of WOT group and its decrement in tissue from DLE group. Yoon et al., 2013, in animals treated with ciprofloxacin and with WSY-1075 herbal formula, showed that the serum levels of TNF-*α* decreased [41]. Here we showed that DLE treatment decreased IFN-*γ* expression in prostate. Penna and coworkers reported that Elocalcitol treatment decreased the production of IFN-*γ* by lymph node cells of autoimmune prostatic mice [7].

IL-6 cytokine is also involved in prostatitis development, increasing the proliferation and inflammatory infiltrate [42]. Chen et al., 2015, observed that higher levels of IL-6 could be associated with the presence of inflammatory cells and prostate hyperproliferation [42]. Some immunomodulating agents like* Serenoa repens* extract (Permixon) decreased IL-6 genetic expression, suggesting that it is associated with decrease of histopathological BPH characteristics [43]. Here, in the DLE treated group we observed a decreased expression of IL-6, a reduction of inflammatory cells, and, even more, a diminishing epithelial cell proliferation. On the other hand, several authors have also suggested that IL-17 possesses an important role in the perpetuation of prostatic inflammation. Penna et al., 2007, using NOD mice model of EAP showed an IL-17 decreased expression in prostate draining lymph node T cells after Elocalcitol treatment. In our work, we observed a tendency to diminish its expression in the gland in DLE treated group, although statistical analysis showed no differences. On the other hand, IL-4 cytokine has been considered to counteract the effects of some proinflammatory molecules such as IFN-*γ*, IL-6, and IL-8, in prostatic inflammation [44]. Some reports on healthy prostatic cells and on BPH cells suggest that the expression of IL-4 diminishes hyperproliferation [36]. Moreover, studies in patients with prostatic cancer have indicated that aspirin, a nonsteroidal anti-inflammatory drug used for prostatic inflammation, is able to increase the serum levels of IL-4 [45]. These results are in concordance with our findings, observing that, in the groups treated with Dexamethasone or DLE, we found an increased expression of IL-4 and a reduction in the expression of some proinflammatory cytokines such as IL-6 and IFN-*γ*.

Vykhovanets et al., 2008, showed that the administration of TNF-*α*, IL-6, or IL-1*β* induces the activation of NF-*κ*B in the prostate and that Dexamethasone could downmodulate the activation of the NF-*κ*B cytokine [46]. A similar result has been showed in an ulcerative colitis model in which Dexamethasone increased the expression of IL-4 [47]. In our study, DLE and Dexamethasone significantly decreased the expression of TNF-*α*, IFN-*γ*, and IL-6 and increased the expression of IL-4. This cytokine behavior is in concordance with the anti-inflammatory effects obtained in an osteoarthritis model, in which DLE inhibited NF-*κ*B activation by diminishing the proinflammatory status [8].

## 5. Conclusion

In conclusion, here we give evidence to support the theory that DLE decreases the inflammatory infiltrate in autoimmune prostatitis and consequently the levels of serum prostatein by decreasing the expression of some proinflammatory molecules, such as CD45, TNF-*α*, IFN-*γ*, and IL-6 and increasing the expression of the anti-inflammatory cytokine IL-4, in a similar way that the Dexamethasone treatment did, but without the undesirable effects [32]. Therefore, we propose DLE as an alternative, adjuvant, or complementary immune-modulator treatment for autoimmune prostatitis and also for the prevention of other complications such as BPH and cancer.
